# A Safe and Multitasking Antimicrobial Decapeptide: The Road from De Novo Design to Structural and Functional Characterization

**DOI:** 10.3390/ijms21186952

**Published:** 2020-09-22

**Authors:** Bruna Agrillo, Yolande T. R. Proroga, Marta Gogliettino, Marco Balestrieri, Rosarita Tatè, Luigi Nicolais, Gianna Palmieri

**Affiliations:** 1 Materias Srl, 80146 Naples, Italy; bruna.agrillo@materias.it (B.A.); nicolais@unina.it (L.N.); 2 Department of Food Microbiology, Istituto Zooprofilattico Sperimentale del Mezzogiorno, 80055 Portici, Italy; proroga.yolande@izsmportici.it; 3 Institute of Biosciences and BioResources, National Research Council (IBBR-CNR), 80131 Napoli, Italy; marta.gogliettino@ibbr.cnr.it (M.G.); marco.balestrieri@ibbr.cnr.it (M.B.); 4 Institute of Genetics and Biophysics, National Research Council (IGB-CNR), 80131 Naples, Italy; rosarita.tate@igb.cnr.it

**Keywords:** antimicrobial peptide, food pathogens, food packaging, antifungal activity, MDR *Salmonella* spp.

## Abstract

Antimicrobial peptides (AMPs) are excellent candidates to fight multi-resistant pathogens worldwide and are considered promising bio-preservatives to control microbial spoilage through food processing. To date, designing de novo AMPs with high therapeutic indexes, low-cost synthesis, high resistance, and bioavailability, remains a challenge. In this study, a novel decapeptide, named RiLK1, was rationally designed starting from the sequence of the previously characterized AMP 1018-K6, with the aim of developing short peptides, and promoting higher selectivity over mammalian cells, antibacterial activity, and structural resistance under different salt, pH, and temperature conditions. Interestingly, RiLK1 displayed a broad-spectrum of bactericidal activity against Gram-positive and Gram-negative bacteria, including multidrug resistant clinical isolates of *Salmonella* species, with Minimal Bactericidal Concentration (MBC) values in low micromolar range, and it was effective even against two fungal pathogens with no evidence of cytotoxicity on human keratinocytes and fibroblasts. Moreover, RiLK1-activated polypropylene films were revealed to efficiently prevent the growth of microbial spoilage, possibly improving the shelf life of fresh food products. These results suggested that de novo designed peptide RiLK1 could be the first candidate for the development of a promising class of decameric and multitask antimicrobial agents to overcome drug-resistance phenomena.

## 1. Introduction

The alarming development and rapid spread of antibiotic resistance among pathogenic microbes has emerged as the major cause of the reduced effectiveness of antimicrobial therapies, thereby representing a huge challenge for modern medicine and a very large public health threat [1,2]. Hence, important measures must be taken in the face of the growing risk of antimicrobial resistance, and it has become strictly urgent and necessary to find innovative strategies aimed at identifying new compounds effective against multidrug-resistant pathogenic microorganisms [3,4].

Over the last years, antimicrobial peptides (AMPs), evolutionary ancient factors of the innate immune system, have attracted increased attention as novel antimicrobials to replace or supplement conventional antibiotics for the control of infections sustained by pathogens, due to the broad-spectrum activity, and unique membrane-action mechanism of these compounds that is mainly related to their amphipathic properties [5,6]. In contrast to most available antibiotics that target specific biosynthetic pathways, one of the primary mode of action explicated by most AMPs involves the interaction with the microbial membranes through Coulombic attraction, leading to pore formation that destroys the membrane integrity and causes cell death [7]. As a result, AMPs are effective against a diverse spectrum of organisms, such as Gram-positive and Gram-negative bacteria, as well as fungi and viruses, and their nonspecific mode of action significantly prevents resistance phenomena, as it is metabolically costly for most microbes to mutate or repair membrane components [8,9]. Despite the promising properties displayed by AMPs, some drawbacks, such as long sequences translating into high production costs, high hemolytic activity, and cytotoxicity as well as a remarkable susceptibility to in vivo enzymatic degradation and salt inactivation, have limited their practical applications [10]. Therefore, a rational in silico analysis to design novel AMPs with optimized structural properties, and/or to project chemical modifications of existing ones, represent a promising strategy to overcome the limitations of native peptides and improve the therapeutic use of AMPs in drug-resistant bacteria or fungi treatments [11]. Indeed, proper changes introduced in the sequence of template peptides, such as truncation and amino acid-selective substitution, can alter their crucial physicochemical parameters, thus, definitely influencing the bactericidal, cytotoxic and anti-biofilm potential of AMPs. This approach allows to get molecules with improved antimicrobial efficacy, broader spectrum of action, and reduced costs of production, correspondingly [12,13].

In a previous study, a 12-residue cathelicidin-related antimicrobial peptide, namely 1018-K6, was developed in silico and deeply characterized [14]. The peptide exhibited a high structural stability and potent antimicrobial, anti-attachment, and anti-eradication biofilm activities at low-micromolar range against both Gram-positive and Gram-negative bacterial species, including methicillin-resistant Staphylococcus aureus (MRSA) and other antibiotic resistant bacteria [14,15]. Microscopy studies evidenced that 1018-K6 did not influence the proliferation nor the morphology of human cell lines compared to the controls [14,16]. From the structural point of view, the peptide adopted a mixed α-helical/-β-sheet conformation in the presence of bacterial membrane mimics and studies on the molecular mechanisms demonstrated that it belongs to the membrane-interacting compounds family [14]. Moreover, 1018-K6 was still active and preserved its excellent antimicrobial and anti-biofilm abilities upon surface immobilization on different kinds of materials such as polyethylene terephthalate (PET) and nanoparticles [16,17]. In light of these considerations, 1018-K6 has been considered a very promising template for the development of a next generation of AMPs.

In this study, a panel of shorter 1018-K6-derived peptides was created *in silico* with the aim to strengthen and maximize broad-spectrum activity against different type of pathogens, improve their stability and safety, and reduce the production costs. The analog peptides were projected by truncating 2-amino-acids at the N-terminus of 1018-K6 and by introducing selective and specific amino acid substitutions in the parental sequence. Considering the relevant effect of the most important physicochemical properties on the antimicrobial activity of AMPs such as length, amino acid composition, total charge, hydrophobicity, hydropathicity, and amphipathicity, the decameric peptide named RiLK1 resulted to be the best derivative that was subjected to the next structural and functional characterizations. Firstly, to investigate the result of the introduced variations on the peptide conformation and membrane association, the fluorescence and Circular Dichroism (CD) spectroscopic analyses in membrane mimicking-models and several environmental conditions, were performed. In parallel, the antibacterial and antifungal activities against some of the most common bacterial pathogens and spoilage fungi worldwide were also evaluated in vitro, together with the salt susceptibility and cytotoxicity toward human cells. Finally, the antimicrobial potential of RiLK1 was also determined when covalently bonded to commercial polypropylene (PP) films, exploring the ability of the projected active packaging to prolong the shelf life of fresh foods, improving their microbial quality and safety.

## 2. Results and Discussion

### 2.1. Design of New AMPs

Antimicrobial peptides based on naturally occurring AMPs are amphiphilic agents with broad-spectrum antimicrobial activities and able to kill pathogens mainly by mechanically damaging the integrity of the bacterial membrane [5,6]. AMPs, therefore, represent the potential alternatives to chemical preservatives to avoid microbial spoilage without causing drug-resistance [18].

Unfortunately, the first-generation of antimicrobial peptides have shown some important limitations resulting in poor commercial perspectives [19,20].

Therefore, to identify a new generation of AMPs it is important to taking in mind essential properties such as lower dosages, higher efficacy, fewer side effects, and, therefore, lower production costs.

Hence, at the first stage, starting from the 1018-K6 sequence, a set of second-generation of short peptides with a length of ten amino acids was generated. Firstly, the removal of two residues and the subsequence single point substitutions were planned in the N-terminal region of 1018-K6 sequence, as it was reported that the substitution of any residues at the positions Val7-Arg12 in the parent peptide (NH2-VRLIVKVRIWRR-CONH2) substantially reduced the antimicrobial activity and were mostly not favorable [21,22]. To this aim, the following criteria were adopted: (i) deletion of a Val residue, as the analysis of amino acid frequency in the AMPs available at CAMP database revealed that this amino acid was less often encountered [23]; (ii) deletion of a further neutral amino acid between Iso or Leu to conserve unaltered the net positive charge, since it is essential for the interactions with the negatively charged membranes of bacteria; (iii) replacement of one neutral amino acid with Trp residue to enhance the total Trp ratio and the hydropathicity, which are key physicochemical parameters influencing the ability of several AMPs to adopt an amphipathic conformation upon binding to the bacterial membrane [24,25]; (iv) conservation of at least an Arg residue, as it has been observed that this amino acid strongly improves the antibacterial activity, due to its enhanced membrane permeability with respect to Lys [26].

Therefore, the reliability of the new set of AMPs, in terms of all the relevant physicochemical properties that are necessary to explicate the antimicrobial activity, was predicted using several software solutions, such as PKePred, PEPlife, APD3, and CAMP. Based on these in silico analyses, the 10-mer peptide named RiLK1, which was characterized by the same net cationic charge (+5) but higher half-life, hydrophobicity, total Trp ratio, amphipathicity, hydropathicity, and Boman index, with respect to the parent 1018-K6, was selected for further biophysical examinations as well as in vitro antimicrobial tests (Table 1).

### 2.2. Conformational Characterization of RiLK1

It has been reported that the interactions of an AMP with membrane components should induce conformational changes of the peptide [27,28]. In this context, to investigate on the conformational behavior of RiLK1 in membrane-mimetic environments, the temporal changes in the secondary structure of the peptide over a period of 24 h were studied by Circular Dichroism (CD) spectroscopy in the presence of SDS, which has been used extensively to mimic the anionic composition of the outer leaflet of the membrane bacteria cells [29].

**Figure 1:**
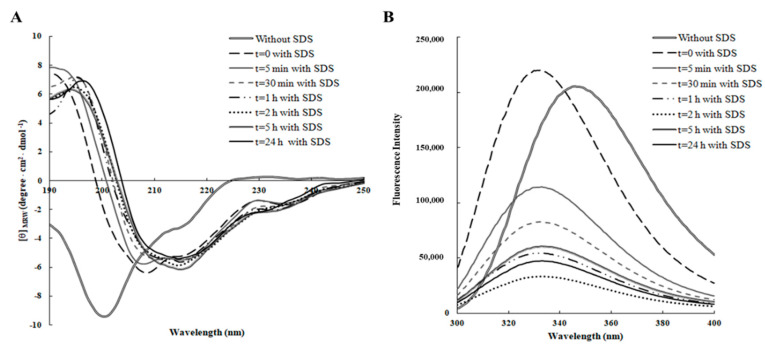
Time-dependent effect of SDS on the secondary and tertiary structure of RiLK1 monitored by spectroscopic analyses. (**A**) Far-UV Circular Dichroism (CD) spectra of the peptide (0.1 mg/mL) were recorded in 10 mM Tris-HCl buffer pH 7.0 in the presence or absence of SDS (3 mM) over the time at 25 °C. (**B**) Intrinsic fluorescence emission spectra of RiLK1 (0.1 mg/mL) in 10 mM Tris-HCl buffer, pH 7.0 in the presence or absence of SDS (3 mM) over the time at 25 °C.

As shown in Figure 1A, RiLK1 exhibited a prevailing disordered structure in aqueous solution only, as revealed by the typical far-UV CD spectrum, which was characterized by a strong negative band below 200 nm and a weak positive band at 230 nm. After addition of SDS, the dichroic spectrum immediately underwent a dramatic shape change resulting in a more ordered structure with the appearance of a negative peak at ~218 nm and a positive peak at ~195 nm, suggesting that RiLK1 adopted predominantly a β-sheet conformation that was well conserved during the time. These findings are consistent with the previous results showing that almost all linear antimicrobial peptides are unstructured in water solution and adopt their active folding when in contact with the biological membranes [30]. To calculate the related contents of the secondary structures of the peptide, the deconvolution of the CD spectra was performed using two different databases and the corresponding results are listed in Table 2. Both analyses confirmed the folding of RiLK1 upon interaction with SDS, evidencing a slight decrease of the α-helical and unordered structure content and a subtle increase of β-structures over experimental time span, revealing a high structural stability of the peptide in the assay conditions.

The conformational studies on RiLK1 were carried out, also, by fluorescence spectroscopy, tacking advance of the presence of two tryptophan residues in the peptide sequence. Indeed, this amino acid is the mainly natural fluorophore in proteins that can be selectively excited at 295 nm, and whose fluorescence is strongly influenced by the polarity of its local microenvironments [31]. Consequently, tryptophan can report on environmental changes during events, such as folding or unfolding and its maximal wavelength (λemi, max), can be used to study the interactions between Trp-containing peptides and membrane-mimetic systems [32]. In aqueous solution, the maximum fluorescence emission was observed at approximately 350 nm that is the typical value for Trp residue when it is fully exposed to a hydrophilic environment (Figure 1B). Immediately after SDS addition (*t* = 0), an increase in the fluorescence intensity and a shift to lower wavelength (“blue shift”) were observed, consistent with a decreased flexibility of Trp residues, which were more sterically confined and a change in the polarity of the microenvironments surrounding the tryptophans, becoming more hydrophobic. Furthermore, a gradual large decrease in the peak fluorescence intensity evidenced by RiLK1 during the time could be indicative of the establishment of strong interactions between the peptide and the negatively charged SDS structures, which partly sequestered the two Trps; thus, resulting in a fluorescence quenching effect. Therefore, these results suggested that the two tryptophan residues in our peptide sequence likely play a critical role in its antimicrobial activity, anchoring the peptide into membranes to drive its permeation.

**Figure 2:**
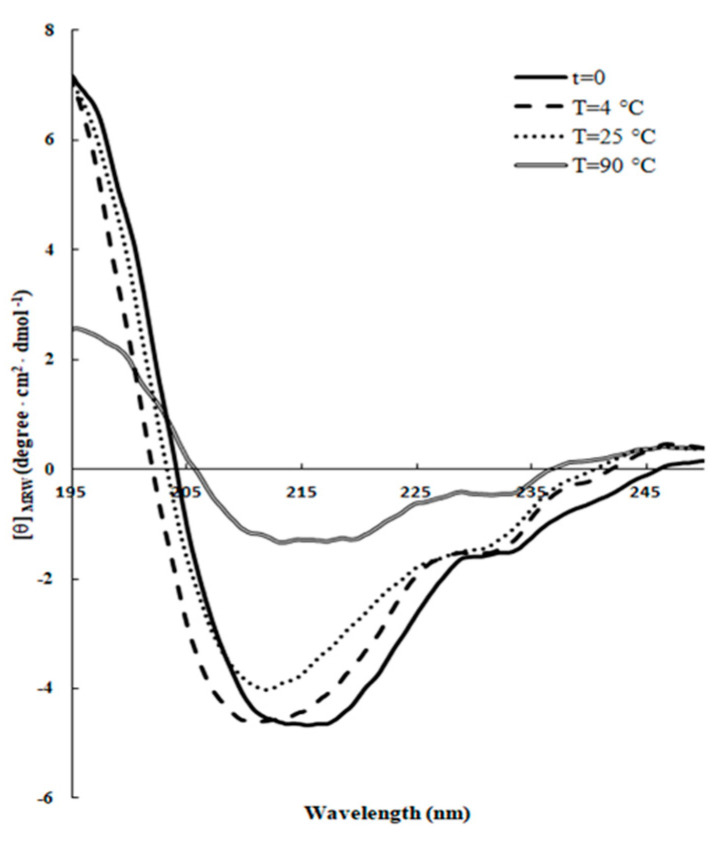
Effect of the temperature on the secondary structure of RiLK1. Far-UV CD spectra of the peptide (0.1 mg/mL) were acquired in 10 mM Tris-HCl buffer pH 7.0 in the presence of 3 mM SDS at three different temperatures (4 °C, 25 °C, and 90 °C) after 24 h incubation.

It has been pointed out that the antibacterial activity of some AMPs is greatly attenuated by certain physical parameters, such as pH, high salt, and high temperature [33]. Therefore, the real efficacy of functional application of these peptides depends on their structural stability in specific environments [31]. To this aim, peptide samples were incubated for 24 h at different temperatures or pH values with SDS and the changes in the secondary structure were analyzed by CD spectroscopy. As shown in Figure 2, the thermal treatment did not induce significant changes in the shape of the spectra, which was consistent with a stable β-sheet folding conformation at the three temperatures investigated, although the β-structures seemed to be less thermally stable at 90 °C, with respect to those at 4 °C and 25 °C after 24 h incubation (Table 3).

As far as the effect of pH is concerned, it is known that the net charge of antimicrobial peptides, which depends by the presence and richness of basic and acidic amino acids, is recognized as playing a key role in their function and an increase in the overall net positive charge leads usually to an improvement in the antimicrobial activity. Therefore, small changes in the pH can interfere with the peptide net charge as each amino acid has an exclusive pKa and isoelectric value.

**Figure 3:**
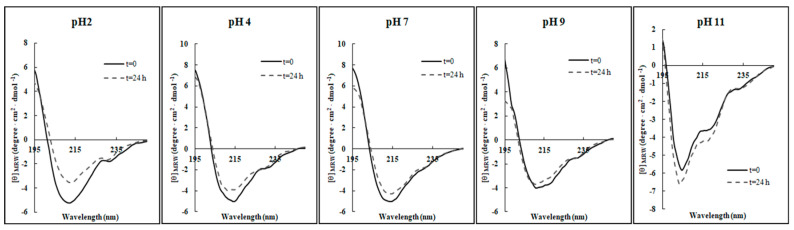
Effect of pH on the secondary structure of RiLK1. Far-UV CD spectra were obtained by incubating the peptide (0.1 mg/mL) in buffers at different pHs for 24 h at 25 °C, and in the presence of SDS at a final concentration of 3 mM.

As depicted in Figure 3, only slight structural perturbations were observed in all the conditions investigated, indicating that in general the pH does not markedly affect the β-sheet integrity of RiLK1 over 24 h incubation in the range investigated, as also evidenced in Table 4. Only at extreme alkaline conditions (pH 11.0), there was a noticeable variation in the CD spectrum of RiLK1, which was more inclined to assume a random-coil intermediate structure.

**Figure 4:**
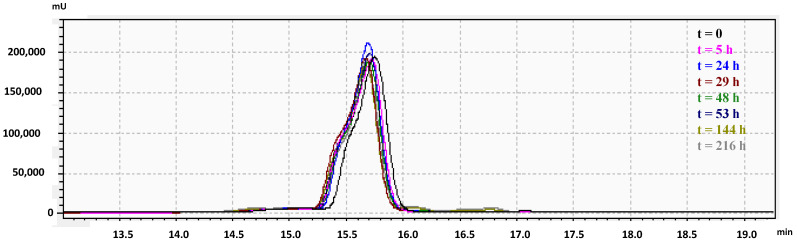
Stability in saline solution of RiLK1 determined by Reverse-Phase High-Performance Liquid Chromatography (RP-HPLC) on a C18 column. The peptide at final concentration of 50 μM was incubated in the presence of NaCl (1M) until 9 day at 25 °C. At each incubation time, the peptide solutions were recovered and analyzed by RP-HPLC. The solution at time 0 (t = 0) was used as control. The chromatograms are representative of three independent experiments.

One of the multiple obstacles to develop AMPs for biotechnological applications is a significantly reduced antibacterial potency in physiological salt conditions [34]. Therefore, salt sensitivity of RiLK1 was evaluated in the presence of 1 M NaCl and its stability in saline solution was quantitatively monitored up to 9 days by Reverse-Phase High-Performance Liquid Chromatography (RP-HPLC) analysis. As shown in the chromatographic profiles reported in Figure 4, no precipitation or aggregation phenomena occurred during the incubation periods in 1 M salt, thus, indicating a high lifetime and stability of the peptide solution over the incubation period, at saline concentrations that are higher than the human physiological values (150 mM), or those typical of some food preservative solutions, such as cheese brine.

### 2.3. In Vitro Antimicrobial Activities of RiLK1

The evaluation of the antibacterial efficacy of RiLK1 was determined against some of the most representatives Gram-positive (*Staphylococcus aureus* and *Listeria monocytogenes* LM3) and Gram-negative (*Escherichia coli* and *Salmonella* Typhimurium) pathogens associated with food poisoning. Since bacteria are remarkably resilient and can adapt rapidly in response to a change in the environment changes increasing their virulence and resistance, all of the strains under investigation were isolated from food products [35].

The antibacterial efficacy of RiLK1 was tested using the kinetic growth-inhibition assay, which represents an alternative dynamic method to the static MIC [36]. Indeed, since this value is determined at a fixed point in time after exposure to drug concentrations that do not change during the entire incubation interval, it is not possible to get many details on how the growth rate of bacteria is affected by the antimicrobial at different concentrations [36].

**Figure 5:**
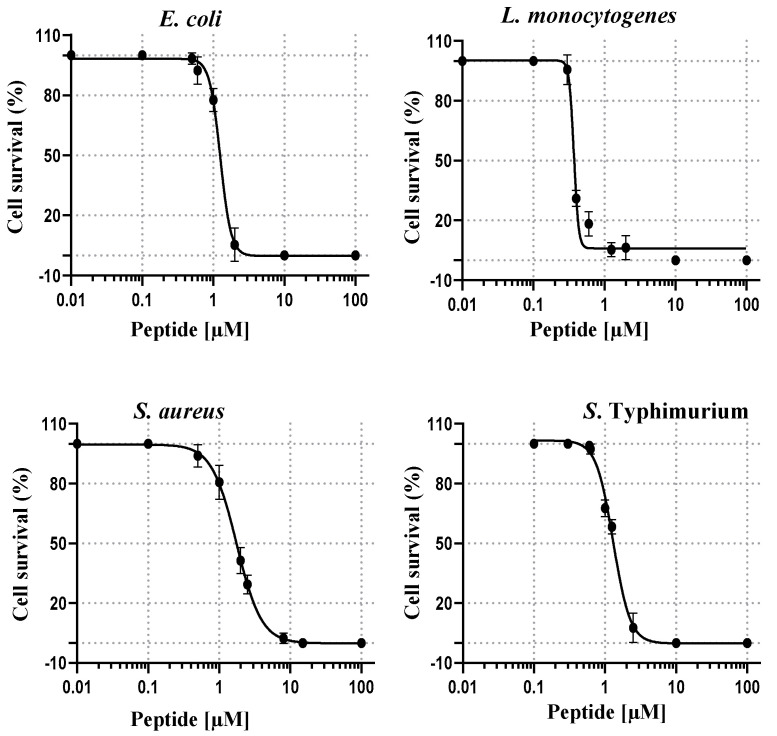
Dose-response effect of RiLK1 on the survival of different foodborne pathogens. Bacteria were incubated in the presence of increasing concentrations of the peptide. Data were determined by enumeration of the surviving colony forming units (CFU) on plates seeded with the pathogen incubated with the different peptide concentrations. Results were expressed as the percentage of colony forming units (CFU) survival, with respect to the colony counted in the control plates. The half-maximal inhibitory concentrations (IC_50_) and Minimal Bactericidal Concentration (MBC) values of the tested peptide against each bacterial strain were calculated using GraphPad Prism version 6.00. Data are presented as means ± standard deviation (s.d.) of three separate experiments performed in triplicate.

As depicted in Figure 5, the sigmoidal dose-response curves of RiLK1 showed a sharp drop in bacterial (expressed as percentage) growth as its concentration was increased, and allowed to determine the half-maximal inhibitory concentrations (IC_50_), which ranged from 0.46 ± 0.01 µM to 1.98 ± 0.25 µM, with respect to those measured for 1018-K6, ranging from 0.30 ± 0.02 µM to 2.30 ± 0.27 µM (Table 5).

Additionally, the bactericidal activity of RiLK1 was evaluated against all the bacteria strains in comparison to the parental peptide 1018-K6 by quantifying the minimum bactericidal concentration (MBC). As shown in Table 5, the short derivative peptide exhibited a bactericidal activity equal to that of 1018-K6 against *S. aureus* (MBC = 16.0 µM) and *E. coli* (MBC = 2.0 µM) strains. On the contrary, RiLK1 displayed a stronger killing efficiency than the parental peptide against *L. monocytogenes* and *S. typhimurium*, with MBC values 4- and 10-times lower than those of 1018-K6, respectively.

**Figure 6:**
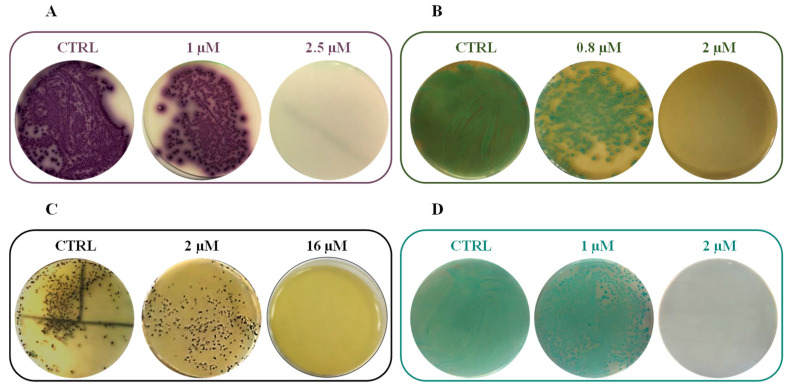
Antibacterial activity of RiLK1 against different foodborne pathogens: (**A**) *S.* Typhimurium, (**B**) *L. monocytogenes*, (**C**) *S. aureus*, and (**D**) *E. coli*. CTRL: each tested pathogen without treatment. Bacterial cultures treated, or not, with different concentrations of peptides were seeded on selective plates. The photographs are representative of three independent experiments performed in triplicate.

Representative plates of RiLK1 against all the tested pathogens were reported in Figure 6.

It is worth noting that one of the most susceptible strain to the RiLK1 action resulted to be *L. monocytogenes* LM3, belonging to the serotype 4b based on the PFGE (Pulsed Field Gel Electrophoresis) profile [14]. This is notable considering that this serotype has been involved in most of reported human listeriosis cases (more than 95%) and displayed strong adaptation or resistance phenomena to antibiotics and disinfectants [37]. Altogether, these findings pointed out a strong inhibitory and bactericidal activity of RiLK1 against all the pathogens tested, confirming its broad spectrum of action. In addition, the replacement and nature of the N-terminal residues in the 1018-K6 sequence, together with the reduction of the chain length effectively enhanced the potency of our lead compound, suggesting that the first six amino acids at the N-terminus can be modified to further improve the antibacterial activity.

In order to check whether the peptide under investigation retained its bactericidal activity against multidrug-resistant strains, preliminary antibacterial experiments were performed using a clinical multidrug-resistant ampicillin, chloramphenicol, streptomycin, sulfonamides, and tetracycline (ACSSuT) isolate of *Salmonella* as the representative strain, which is considered one of the major zoonotic and human pathogens worldwide [38]. Remarkably, the findings demonstrated that RiLK1 exerted potent killing effects against the clinical isolate (MBC = 1.25 µM), two-fold lower than those observed against the strain isolated from food products and those determined with the parental peptide 1018-K6 against ACSSuT strain, thus, suggesting that RiLK1 might have a high potential also in the medical field.

### 2.4. In Vitro Antifungal Activities of RiLK1

The incidence of serious infections caused by pathogenic fungi that are resistant to the commonly used antifungal drugs is increasing dramatically, and the development of new antifungal agents is becoming an urgent need to avoid a global collapse in the ability to control this type of infections at different levels ranging from human health to food security [39,40].

For a long time, besides altering food properties, fungi were not considered as particularly harmful to human health and it is only in recent periods that several mycotoxin-producing fungi have been considered as a major threat to human and animal health, being responsible for different adverse effects [41]. Therefore, industrials and scientists are looking for efficient solutions to avoid and/or control the fungal spoilage and their associated infections. Among the 40 fungal species usually considered as pathogens, *Aspergillus* spp. and *Candida* spp. represent two of most relevant genera of mold and yeasts, which are responsible for the majority of fungal infections [40].

**Figure 7:**
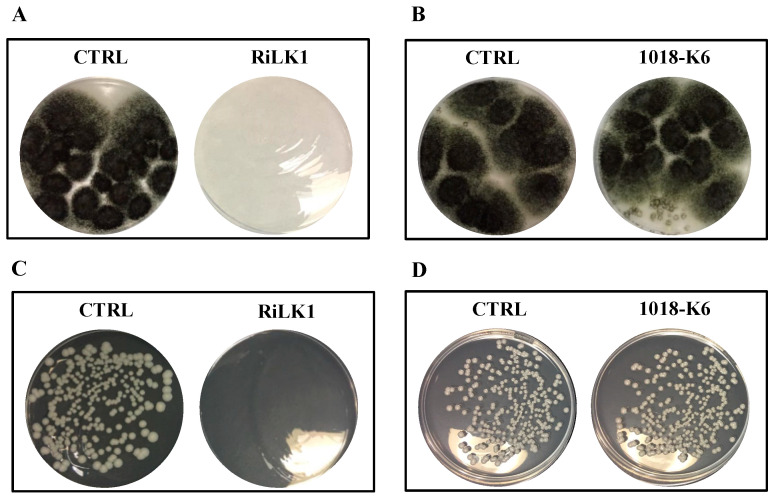
Antifungal activity of RiLK1 and 1018-K6 against pathogenic fungi. (**A**,**B**) *Aspergillus brasiliensis* and (**C**,**D**) *Candida albicans*. The fungal cultures untreated (CTRL) or treated with the peptides at 25 µM concentration were seeded on DG18 (Dichloran 18% Glycerol Agar) plates. The photographs are representative of three independent experiments performed in triplicate.

In this study, to preliminary investigate whether RiLK1 possessed antifungal activity, the effects of the peptide on the growth of the two reference fungal strains *C. albicans* ATCC 14053 and *A. brasiliensis* ATCC 9341, were investigated in comparison with 1018-K6. Notably, susceptibility testing clearly showed that RiLK1 was very effective, being able to inhibit 100% growth of both fungi at 25 μM concentration (MFC), whereas the parental peptide 1018-K6 exerted no detectable antifungal activities even at the highest concentration (50 μM) used in the assay (Figure 7).

It can be argued that the different inhibitory activity of 1018-K6 across microbial species could be attributed to the differences in the cell membrane composition displayed by bacteria and fungi.

Altogether, these results further emphasized the effectiveness of the modifications introduced in the sequence of 1018-K6 both in terms of chain length and nature of the residues. Indeed, the 10-mer peptide RiLK1 exhibited excellent antibacterial and antifungal activities in contrast to its parent, thus offering important advantages in a lot of applications as it would be helpful to have a single agent able to treat bacterial and fungal co-infections, including those caused by pathogens that are resistant to currently available drugs.

### 2.5. Evaluation of Detrimental Effects of RiLK1 on Mammalian Cells 

**Figure 8:**
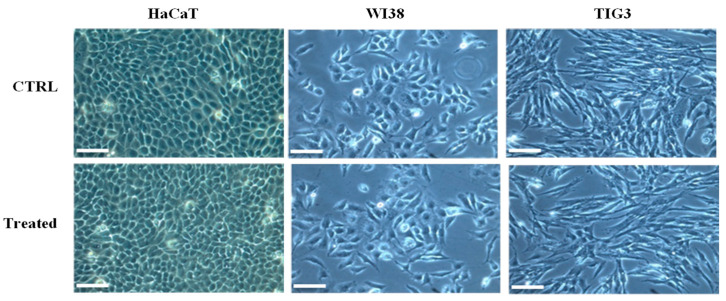
Morphological observation of different human cell lines treated with RiLK1 under phase-contrast microscope. Keratinocyte (HaCAT), embryonic (WI38), and fetal (TIG3) lung fibroblastic cell lines were incubated at 37 °C for 24 h in absence (CTRL) or in presence (treated) of RiLK1 at the maximum concentration tested (10 µM). The microscope images are representative of three independent experiments performed in triplicate. Bar is equal 100 µm.

The inherent risks of the use of antimicrobial agents, which include the determination of cytotoxicity towards human cells, should be addressed in order to consider them for practical applications, specifically in the medical field and food safety [42]. In this study, the potential of RiLK1 to affect the cell morphology was evaluated against human keratinocytes (HaCAT), and fetal (WI-38) and adult (TIG-3) lung fibroblast-like cell lines, by incubating the cells with increasing concentrations (1−10 µM) of RiLK1 for 24 h followed by light microscopy. As shown in the micrographs reported in Figure 8, RiLK1 did not induce any change or alterations in the morphology of all the three human cells, similarly to that already observed with the parent 1018-K6 (Supplementary Figure S1) [14].

Indeed, the same experiments were performed on WI-38 and TIG-3 cell lines exposed to 1018-K6 at the standard concentration of 10 µM. The obtained results demonstrated that the morphology of both cell lines of interest was not affected by the treatment with the parent peptide (Supplementary Figure S1).

Moreover, the cytotoxic potential of RiLK1 on mammalian cells was initially investigated in vitro by Neutral Red Uptake (NRU) assay using the mammalian BALB 3T3 clone A31 fibroblast cell line. Following the treatment with increased concentrations of RiLK1 (10, 25, and 50 µM), it was observed that the cell viability was recorded as 98%, 98.7%, and 98.9%, respectively, which was calculated by using the equation (1) reported in Material and Methods. Therefore, these findings demonstrated that RiLK1 did not exert any cytotoxic effects against the mammalian cells under investigation at the tested concentrations, even at the highest concentration employed (50 µM), which was sufficiently high to kill all the target pathogen bacteria.

It is noteworthy that although both fungi and host cells are eukaryotic organisms, RiLK1 showed high selectivity towards fungi over mammalian cells, possibly attributable to the different membrane lipid components in this kind of cells. Moreover, the significant differences in the composition of eukaryotic membranes in comparison to prokaryotic membranes could highlight the important selectivity of RiLK1 for microbial cells. Indeed, the cationic peptide will preferentially bind to the negatively charged phospholipid bilayer of bacterial cells rather than to that overall neutrally charged in eukaryotic [43]. This is advantageous with regard to wider potential use of RiLK1 for biotechnological and clinical applications.

### 2.6. Functionalization of PP Polymer with RiLK1

Currently, food preservation, quality maintenance, and safety are major concerns of the food industry [44]. Indeed, while the deterioration by spoilage microorganisms strongly affects the food shelf life, the growth of pathogenic microorganisms represent a danger to public health [45]. Therefore, controlling the growth of these microorganisms is necessary to reach a high level of food quality, freshness, and safety. In this scenario, the antimicrobial packaging has been considered one of the most promising innovations among the active packaging technologies that could play a role in increasing the food shelf life and reduce the risk of microbial contamination [44,46]. As a result, researchers have shown a notable interest in developing new antimicrobial peptides with bioactive properties, which can be incorporated into polymeric materials to create innovative antimicrobial packaging.

In this work, the de novo-designed AMP RiLK1 was immobilized on polypropylene (PP) films to get an advanced antimicrobial packaging material, whose bio-preservative efficacy was evaluated by detecting the growth of spoilage microorganisms responsible for product lost in perishable foods. To this aim, commercial PP films, pre-activated by double side corona discharge treatment to promote their adhesion performances, have been chosen to allow the covalent binding with the antimicrobial peptide. The covalent attachment of RiLK1 on pre-activated PP films was executed by a one-phase immobilization process involving the simple immersion of the polymeric surfaces in a DMSO solution of the peptide for different contact times in the presence of molecular sieves which were used as a drying agent to sequester H_2_O product and drive the coupling reaction. Direct conjugation of AMP to the films is favored by the formation of amide bonds between the reactive carboxylic acid groups (-COOH*) generated on PP by corona and the amine groups of the peptide.

**Figure 9:**
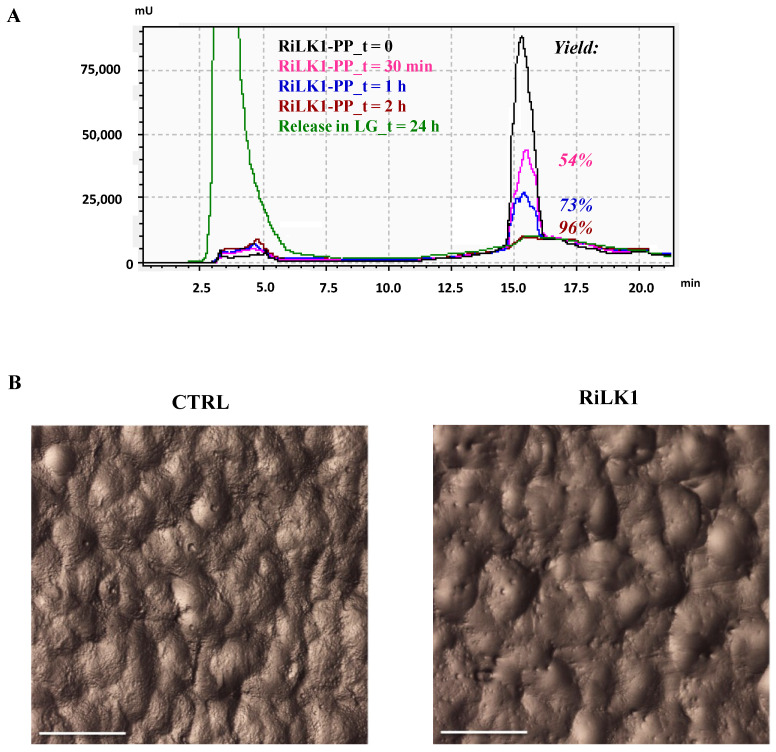
Covalent immobilization of RiLK1 on polypropylene (PP) disks. (**A**) Time course of the coupling reactions of RiLK1 on PP surface determined by RP-HPLC on μBondapak C18 column for the immobilization yield (%) measurement. The PP disks, pre-activated by corona treatment, were incubated for different times in a solution of RiLK1 (50 µM) in DMSO in presence of molecular sieves. After each coupling reaction, the solutions were recovered and analyzed by RP-HPLC. A reference sample (t = 0) prepared with the same peptide solution placed in contact with PP films without adding the molecular sieves was used as control. The chromatogram is representative of three independent experiments. The chromatographic profile in green represents the release analysis of RiLK1 from functionalized PP disks performed by RP-HPLC on C18 column after 24 h incubation at 4 °C in mozzarella brine. A solution of RiLK1 (50 μM) was used as control. (**B**) Stereomicroscope photographs of PP disks without (CTRL) and with the peptide (RiLK1) after the immobilization procedure. The microscope images are representative of three independent experiments performed in triplicate. Bar is equal 0.5 mm.

With the aim to assess the success of our conjugation procedure, the immobilization efficiency of RiLK1 on the polymeric films was quantitatively estimated by RP-HPLC. In this experiment, once the conjugation reaction was completed, the supernatant solutions were recovered at the different contact times and analyzed by RP-HPLC. Specifically, the amount of the peptide attached to the PP disks was indirectly calculated by comparing the peak area of the peptide not bound to the surface at the end of the coupling reaction, with that of the peptide placed in contact with the PP films, but in the absence of the molecular sieves, which are necessary to promote the reaction. As shown in Figure 9A, the coupling yield varied from 50% after 30 min of incubation to 96% after 2 h, which resulted to be the best reaction time. Indeed, a further increase in the contact time between the peptide and the PP films over 24 h did not produce any additional improvement of the immobilization efficiency. To support the chromatographic data on the successful of the surface functionalization technique for PP using the short peptide, a stereomicroscope investigation was performed on the treated and untreated PP disks to quantitatively detect the surface coverage and morphology (Figure 9B). As shown in the microscope pictures, RiLK1 was effectively bound to the surface with an optimal coverage after the covalent attachment. In addition, analyses of the surfaces’ physical composition showed obvious and strong differences in terms of roughness of the materials being examined. Specifically, the presence of the bound peptide reduced the roughness surface geometry typical of the corona activated PP polymers, further confirming the complete covering of the polymeric surfaces after the conjugation reaction.

### 2.7. Effect of Antimicrobial Packaging on Mozzarella Cheese

Generally, investigation on the efficiency analysis of an antimicrobial packaging system should be conducted using real food products instead of artificial experimental conditions, such as culture broth or agar media [45]. For these reasons, the antimicrobial effectiveness of our developed active system RiLK1-PP was tested on samples of mozzarella cheese, which represents one of the greatest popular fresh dairy products in Italy. As for most of the perishable products, the short microbial stability greatly affects the shelf life to a few days and, consequently, its diffusion on the local market. Therefore, the use of active packaging during the storage could allow extending the shelf life while rendering inactive foodborne microorganisms. First, an important requisite for an active food packaging is to ensure that the bioactive compound will not migrate to the food, thus offering the advantage of not requiring approval as a food additive. For this reason, the stability of covalently bonded RiLK1 on PP under simulated storage conditions was verified. Specifically, the RiLK1-PP films were immersed and stored in mozzarella cheese brine for 24 h at 4 °C, and then the potential release of the peptide was monitored by RP-HPLC, using the free RiLK1 at the concentration employed for the functionalization as control.

**Figure 10:**
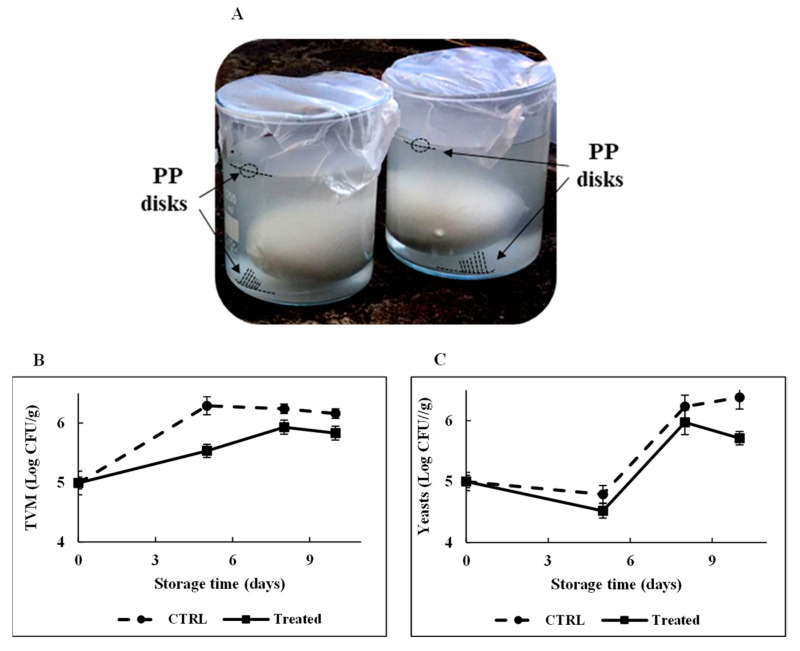
Effect of RiLK1-PPs on the microbiological quality of mozzarella cheese. (**A**) Photograph of preparation of mozzarella samples for the shelf life testing. A total of 50 g of fresh mozzarella cheese were immersed in 200 mL of brine in the presence of two functionalized PP disks placed above and below the food product. Not-functionalized PP disks were used as control. PP disks are indicated by the arrows. Effect of RiLK1-PPs treatment on (**B**) Total Viable Microorganism (TVM) and (**C**) yeast counts of mozzarella cheese samples during the storage time. CTRL: not-functionalized PP disks; treated: RiLK1-PP disks. Data are presented as means ± s.d. of five different samples analyzed in triplicate.

It is pointed out that the choice to perform the stability experiments for a time interval of 24 h was based on the consideration that, in food packaging applications, the possibility to inhibit microorganism proliferation for some hours already represents a striking result for processed food with a short shelf life. As shown in the chromatogram highlighted in red in Figure 9A, no peptide-release process occurred from the functionalized polymeric support during the 24 h incubation, confirming the stable attachment of RiLK1 on the polymers via the covalent coupling, which not allows the peptide to withdraw from the surface. Therefore, the microbiological quality of mozzarella was monitored during the time to assess the antimicrobial effects of RiLK1-PP surfaces by developing an experimental system that reproduced the real condition of storage of the dairy product (Figure 10A). Generally, Total Viable Microorganism (TVM) and mold and yeast counts are used by the food industry as indicators of processing hygiene, storage quality, and potential shelf life [47]. Changes in TVM (Log CFU/g) in mozzarella cheese packaged with the active and control films during 10 days of storage are shown in Figure 10B. TVM of control samples raised of about 1.2 Log CFU/g after 10 days of storage at 4 °C, whereas the increase was 0.8 Log CFU/g at the end of the entire period of observation in mozzarella samples packaged with the active films.

Similarly, the lowest mold and yeast values were observed during the storage in samples stuffed between the two active films, while the highest counts were obtained with mozzarella packed in control disks (Figure 10C). In addition, the overall visual quality of the mozzarella cheese samples packaged in the presence of antimicrobial polymers was preserved, with respect to those stored with control films.

It should be pointed out that these results were not obvious on the basis of two main considerations: (i) the antimicrobial packaging could be ineffective in reducing microbial populations when applied under real conditions due to the chemical complex composition of the selected food which can inactivate the biocidal; and (ii) RiLK1 could not retain its antimicrobial activity when bound to the polymer. Indeed, it is well-known that the ability for free AMPs of drilling a hole on bacterial membranes originates from their freedom in solution and their tendency to assume the correct folding when in the proximity of the membrane. Based on these observations, unlike the soluble counterpart, the immobilization process could restrict the conformational freedom of RiLK1 and influence its orientation. Moreover, the short length of the amino acid chain might not allow it to fully extend into the bacterial inner cell membrane and create a hole. Therefore, it can be hypothesized that immobilization did not influence the folding of the peptide, and the charge-charge interaction should play a dominant role in bacteria killing due to the limited mobility of RiLK1.

## 3. Materials and Methods

### 3.1. Synthesis and In Silico Design of RiLK1

The peptide RiLK1 used in this work was purchased from GenScript Biotech (Leiden, Netherlands). RiLK1 was stored as a lyophilized powder at −20 °C. Prior to experimentation, fresh solutions in 100% DMSO were prepared, briefly vortexed, and sonicated, and these samples were used as stock solutions in all experiments. The following web server and software were used for determining the main RiLK1 features such as the reliability, stability, half-life in vivo, and all the relevant physicochemical properties (Boman index, total net charge, GRAVY index, hydrophobicity, amphipathicity, hydropathicity, aliphatic index, and instability index): PlifePred (PPred) [48], PEPlife [49], Antimicrobial Peptide Database3 (APD3) [50], and Collection of Anti-Microbial Peptides (CAMP) [51].

### 3.2. Bacterial Strains

*Escherichia coli*, *Salmonella* Typhimurium, *Listeria monocytogenes* LM3 (serotype 4b), and *Staphylococcus aureus* were isolated from food products. They were detected in the Laboratory of Microbiological Food Control—Department of Food Microbiology of the Istituto Zooprofilattico Sperimentale del Mezzogiorno in Portici (Naples, Italy) in raw and processed foodstuffs of animal origin. *S*. Typhimurium was isolated from chicken according to UNI EN ISO 6579-1, and it was serotyped according to ISO/TR 6579-3:2014 by agglutination with specific anti-sera for O (State Serum Institute–DK) and H antigens (Difco, Franklin Lakes, NJ, USA), using two strains of *Salmonella enterica* (*S*. Typhimurium and *S*. Blockley), which were kindly provided by the National Reference Laboratory for *Salmonella* (Istituto Zooprofilattico Sperimentale delle Venezie, Padova, Italy) as quality control. *L. monocytogenes* was isolated from fish according to UNI EN ISO 11290-1, *E. coli* was isolated from mussels according to UNI EN ISO 16649-3, and *S. aureus* was isolated from pastry product according to UNI EN ISO 6888-2.

*Salmonella* strains of human origin were collected from hospitals located in Campania region by the Centro Tipizzazione Salmonelle—Department of Food Microbiology of the Istituto Zooprofilattico Sperimentale del Mezzogiorno in Portici (Naples, Italy), which is the local reference laboratory for *Salmonella* serotyping [52]. The strains were isolated from stools of patients with gastroenteritis. The antimicrobial susceptibility was performed by the disk-diffusion method, following the Clinical and Laboratory Standards Institute (CLSI) recommendations. The following antibiotics (Oxoid, Basingstoke, England, and Becton Dickinson, Mississauga, ON, Canada) were used: nalidixic acid (NAL, 30 μg), ampicillin (AMP, 10 μg), chloramphenicol (CHL, 30 μg), gentamicin (GEN, 10 μg), tetracycline (TET, 30 μg), trimethoprim-sulfamethoxazole (SXT, 25 μg), ciprofloxacin (CIP, 5 μg), colistin sulfate (CST, 10 μg), ceftazidime (CAZ, 10 μg), and cefotaxime (CTX, 30 μg). A quality-control strain (*Escherichia coli* ATCC 25922) was included in the test. The antibiotic resistance or susceptibility interpretation was performed according to the CLSI standards. Specifically, the strains displaying intermediate resistance were evaluated as resistant, while those resistant to at least three antibiotic classes were considered multidrug resistant (MDR)

### 3.3. Antimicrobial Assays

The Minimum Bactericidal Concentration (MBC) were determined by the standard broth microdilution method in accordance with the Clinical & Laboratory Standards Institute guidelines (CLSI, 2015). For microbroth dilution assay, *L. monocytogenes*, *E coli*, *S. aureus*, and *S*. Typhimurium were grown in BPW (Thermo Fisher, Milan, Italy). Bacterial cells were cultured at 37 °C in the appropriate culture media until collection and then diluted in fresh broth to final concentration of 1.0 × 10^3^ CFU/mL (CFU, colony forming units). Thereafter, serial dilutions of RiLK1 in the suitable medium (ranging from 1 to 100 μM), prepared starting from a stock solution in DMSO, were added to each bacterial suspension and incubated at 37 °C for 6 h. Samples containing only cell suspension and DMSO were used as controls. MIC is defined as the lowest peptide concentration, which inhibits the visible growth of bacteria. The MBCs were determined by transferring onto selective agar plates (*L. monocytogenes*, Agar Listeria acc. to Ottaviani & Agosti (ALOA) —Biolife Italiana; *S*. Typhimurium, Salmonella Chromogenic agar—Oxoid UK; *S. aureus*, Baird Parker agar base—Biolife Italiana; *E. coli*, TBX agar—Biolife Italiana) 50 μL of the bacterial cell suspensions, taken based on the MICs and incubated 24/48 h at 37 °C for *L. monocytogenes*, *S.* Typhimurium and *S. aureus* while *E.coli* was incubated overnight at 44 °C. MBC is defined as the lowest concentration of peptide at which more than 99.9% of the bacterial cells are killed. IC_50_ is defined as the concentration of a compound that inhibits 50% growth of bacterial cultures relative to control. IC_50_ and MBC values were assessed by GraphPad Prism version 6.00 (Graph-Pad Software, La Jolla, CA, USA). All values were calculated as mean of three independent experiments conducted in triplicate.

### 3.4. Antifungal Assays

Fungal strains used in this study were purchased from the American Type Culture Collection (ATCC, Manassas, VA, USA) as follows: *Aspergillus brasiliensis* ATCC 9341 and *Candida albicans* ATCC 14053 strains. Briefly, for both fungal species, the cell suspension was adjusted to 1.0 × 10^5^ CFU/mL in buffered peptone water (BPW) (bioMerieux, Florence, Italy). Peptide stock solution in DMSO was added to the fungal suspension at a final concentration of 25 µM and 50 µM and incubated for 6 h a 37 °C. The minimum fungicidal concentration (MFC) was determined by plating 100 µL cultures on DG18 plates (Dichloran 18% Glycerol Agar—ISO 21527-2) for CFU counting. After incubation at 25 °C for 7 days, the MFC was defined as the lowest peptide concentration that resulted in 99.9% killing compared with the drug-free group. The analyses were performed in triplicate on three different experiments.

### 3.5. Circular Dichroism (CD) Spectroscopy 

The secondary structure of RiLK1 was investigated by CD spectroscopy. All spectra were recorded on JASCO J-810 spectropolarimeter (JASCO, Tokyo, Japan) equipped with a temperature control unit using a 0.1-cm path-length quartz cell (Hellma Analytics, Milan, Italy) in the 195 nm–250 nm wavelength range at 20 nm/min scanning speed by averaging 5 scans. The CD spectra of the peptides (0.1 g/L) were obtained in different environments in the presence of 3 mM SDS, mimicking the biological membrane, and recorded after 24 h incubation. The effect of pH on the secondary structure of the peptide was evaluated by dissolving the sample in different buffer solutions at 10 mM concentration: glycine-HCl, pH 2.0; sodium acetate, pH 4.0; Tris-HCl, pH 7.0; glycine-NaOH, pH 9.0 and 11.0. Structural changes upon temperature were also recorded in 10 mM Tris-HCl buffer pH 7.0 and 3 mM SDS at three different temperatures: 4 °C, 25 °C, and 90 °C. The folding kinetic measurements of the peptide were carried out in 10 mM Tris-HCl buffer, pH 7.0, in the presence of SDS (3 mM) over 24 h incubations. A blank spectrum of a sample containing all components except the peptide was acquired to baseline-correction of the CD spectra of the peptide. After noise correction, ellipticities were converted to mean residue molar ellipticities [θ] in units of deg cm^2^ dmol^−1^. Secondary structure content was estimated by BeStSel web server [53] and DichroWeb software using CONTIN-LL algorithm [54].

### 3.6. Fluorescence Spectroscopy

The fluorescence emission spectra of the Trp residue in the peptide sequence (0.1 mg/mL) were monitored in 10 mM sodium acetate buffer pH 4.0 in the presence of 3 mM SDS over 24 h incubation at 25 °C by using a Shimadzu RF-6000 spectrofluorometer (Kyoto, Japan). Fluorescence was measured at an excitation wavelength of 280 nm and an emission wavelength ranging from 300 to 400 nm, setting the slit widths at 5 nm.

### 3.7. Ionic Strength Stability

The effect of ionic strength on the peptide stability was examined by reverse-phase (RP) High-Performance Liquid Chromatography (HPLC) analysis. The antimicrobial molecule at a final concentration of 50 µM was incubated in a water solution containing 1 M sodium chloride until 9 days at 25 °C. For the analyses, 200 µL of the sample solution were recovered at different times and loaded onto a µBondapak C18 reverse-phase column (3.9 mm × 300 mm, Waters Corp., Milford, MA, USA) connected to a HPLC system (Shimadzu, Milan, Italy) using a linear gradient of 0.1% trifluoroacetic acid (TFA) in acetonitrile from 5 to 95%. A reference solution at time 0 (t = 0) was prepared under the same reaction conditions and run in parallel. The peptide stability in saline solution was evaluated by comparing the peak area of the peptide at the different incubation times, with that of the peptide at t = 0.

### 3.8. Evaluation of Cell Morphology

The in vitro effect of RiLK1 on the morphology of eukaryotic cells was evaluated against the human keratinocytes (HaCAT), fetal (WI-38) and adult (TIG-3) lung fibroblast-like cell lines purchased from the American Type Culture Collection (ATCC). The human cells were grown in Dulbecco’s modified Eagle’s medium (DMEM) (DIFCO) supplemented with 1% L-glutamine and 10% fetal calf serum (FCS, DIFCO). Cells were seeded in six-well plates (5 × 10^4^ cell/well) and incubated for 16 h at 37 °C in a humidified atmosphere of 5% CO_2_. Then, the cells were treated with different concentrations of peptide (ranging from 1 to 10 μM) for 24 h. Untreated cells were used as negative controls. After 24 h treatment, the cells were washed with Phosphate-Buffered Saline (PBS) buffer, fixed with 4% paraformaldehyde (PFA) for 30 minutes and visualized by phase-contrast microscopy using the DMI6000B inverted fully automated microscope with DFC420 RGB camera (Leica Microsystems, Wetzlar, Germany). Leica LASV5.4 software was utilized for the image acquisition/elaboration (contrast/γ adjusting).

### 3.9. In Vitro Cytotoxicity Assays

Toxicity of RiLK1 on mammalian fibroblasts BALB 3T3 clone A31 (ATCC CCL-163), at increasing peptide concentrations, was determined using the Neutral Red Uptake (NRU) assay. BALB 3T3 clone A31 (ATCC CCL-163) cells were cultivated in Dulbecco’s Modified Eagle’s Medium (DMEM) supplemented with 10% Newborn Calf Serum and 4 mM Glutamine. For the NRU assay, the cells were seeded in 96-well microtiter plate (Thermo Fisher Scientific, Milan, Italy) and incubated for 24 h at 37 °C and 5% CO_2_ in a humidified environment allowing cell sedimentation and the constitution of a subconfluent monolayer prior to treatment with RiLK1. Therefore, cells were exposed at increasing concentrations of RiLK1 (10, 25, and 50 µM) for 24 h at 37 °C. Following treatment, each well was rinsed with 150 μL of D-PBS with Ca^2+^/Mg^2+^ and treated with 50 μg/mL Neutral Red (NR) dye solution for 3 h at 37 °C. Afterwards, each well was again rinsed with 150 μL of D-PBS with Ca^2+^/Mg^2+^ and 150 μL of NR desorb solution (49% ddH_2_O, 50% ethanol, 1% acetic acid) were added. Plates were placed under gentle agitation in darkness for 10 min and the Optical Density of the NR extract at 540 nm was measured spectrophotometrically. Cell viability was expressed as percentage of BALB 3T3 clone A31 cells grown in the treatment medium (DMEM with 5% NCS, 4 mM Glutamine, 0.1% DMSO, and RiLK1), with respect to the control group represented by the cells grown in DMEM with 5% NCS, 4 mM Glutamine and 0.1% DMSO (viability control cells = 100%).

(OD treated cells − OD blank)/(OD Control Cells − OD blank) × 100(1)

Interpretation of the data was performed according to ISO 10993-5:2009: the compound was considered cytotoxic if the relative cell viability of the sample was <70% of the control group, while it was considered non-cytotoxic if cell viability of the sample was ≥70% of the control group.

### 3.10. RiLK1 Immobilization on Polymer Surfaces

Commercial Polypropylene (PP) films, pre-activated by corona treatment on double side, were purchased from Cope Plastics (Melzo, Milano, Italy). The flat surfaces were cleaned twice with ethanol and dried prior to use for chemical reaction. The PP films were cut into disks of 5 cm diameter (surface of 19.6 cm^2^) and immersed for different time intervals at 25 °C in a 100% DMSO solution containing RiLK1, at 50 µM concentration, and 10 pieces/ml of freshly activated molecular sieves (Merck, Italy), with a pore diameter of 4 Å, to allow the continuous removal of the water molecules produced during the coupling reaction. After incubation, the solution was recovered and RiLK1-PP surfaces were cleaned by washing thoroughly in deionized water to remove all the unbound peptide.

### 3.11. Coupling Yield Analysis

The surface concentration of the covalently attached RiLK1 was determined by RP-HPLC analysis. Briefly, 200 µl solution recovered at each time intervals after the coupling reaction, were injected on a Waters µBondapak C18 reverse phase column (3.9 mm × 300 mm) connected to a HPLC system (Shimadzu, Milan, Italy), using a linear gradient of 0.1% TFA in acetonitrile from 5 to 95%. A reference sample was prepared with the same peptide solution placed in contact with PP films without adding molecular sieves necessary to induce the coupling reaction. The amount of immobilized RiLK1 (expressed as a percentage) was indirectly calculated by comparing the peak area of the reference solution and that of the peptide not bound to the surface at the end of the functionalization. All measurements were taken in triplicate.

### 3.12. Microscopy Analysis of RiLK1 Immobilized on PP Disks

To correlate the presence of covalently attached RiLK1 on PP disks and the surface geometry, PP disks untreated and treated with RiLK1 were observed by using a Leica MZ16-FA stereomicroscope (Wetzlar, Germany). Leica EZ software was used to perform the acquisition/elaborations of imagines.

### 3.13. Chromatography Determination of RiLK1 Release from PP Films

After the covalent immobilization reaction, RiLK1-PPs disks were extensively cleaned by washing thoroughly in deionized water and then left into pure water at 25 °C or governing liquid from mozzarella cheese at 4 °C for 24 h. After incubation, a sample (200 µL) of solution was picked up and injected onto µBondapak C18 column to determine the peak area, using a linear gradient of 0.1% TFA in acetonitrile from 5 to 95%. The peptide release was estimated by comparing the peak area of the solution after 24 h incubation with that measured for a reference solution of RiLK1 (50 µM) (t = 0). All analyses were performed in triplicate on three different preparations.

### 3.14. Shelf Life Testing on Mozzarella Cheese

All analyses were conducted on 50 g of buffalo mozzarella cheese purchased from a local market. Briefly, the samples were immersed in 200 mL of governing liquid inside a glass beaker, in contact with two RiLK1-PPs disks of 5 cm diameter (surface of 19.6 cm^2^), which were placed above and below the food product. Not-functionalized PP disks were used as controls. All of the samples were stored at 4 °C ± 3 °C and the microbiological analyses were performed at 0, 5, 8, and 10 days. At each time point, 10 g of mozzarella cheese were aseptically removed from each package and transferred into a sterile stomacher bag with 90 mL of buffered Peptone Water. The mix was homogenized for 3 min at 230 rpm using a peristaltic homogenizer (BagMixer^®^ 400 P, Interscience, Saint Nom, France). After further 10-fold dilutions, the homogenate was spread on Plate Count Agar (PCA) (Biolife, Milan, Italy) or Dichloran Rose Bengal Chloramphenicol (DRBC) Agar plates, and incubated at 30 ± 1 °C for 72 h (ISO 4833-2) or at 25 ±1 °C for 5/7 days (ISO 21527-1), to enumerate Total Viable Microorganism (TVM) and yeasts and molds, respectively. The analyses were performed in triplicate on three different preparations, and the data were expressed as means ± s.d. Results were expressed as Log_10_ Colony Forming Unit (CFU) per gram fresh weight.

## 4. Conclusions

In this study, to develop new weapon solutions against pathogens, a new decapeptide named RiLK1 was de novo designed by specifically modifying the antimicrobial peptide 1018-K6. RiLK1 was highly effective against both fungi and Gram-positive and -negative bacteria, including clinically isolate strains of *Salmonella*, and it retained its structural stability under a range of physical conditions, such as pH, temperature, and high salt concentrations. Moreover, suitable modifications were introduced in the primary sequence of 1018-K6, resulting in an improvement of the microbial selectivity without causing human cell cytotoxicity. Finally, RiLK1 was successfully conjugated to a model polymeric surface without using a spacer. The preliminary in vivo test confirmed the antimicrobial effects of the developed antimicrobial packaging on mozzarella spoilage, possibly extending the quality and safety of the fresh dairy product and suggesting its potential for application in food packaging.

Collectively, our findings strongly support that projected RiLK1 holds potential as a promising candidate for the development of a novel class of decameric and multitask antimicrobial agents for further biotechnological and clinical applications, helping to overcome serious problems that prevent the use of AMPs, such as low stability, toxicity to mammalian cells, high cost of production, and induction of antibiotic-resistance phenomena.

## 5. Patents

Application No. 102017000080068 Publication Date: 14/07/2017. “Peptidi antimicrobici”. Balestrieri M., Palmieri G., Neglia G., Anastasio A., Capuano F., de Stefano L., Nicolais L. Granting date 11/10/2019.

## Figures and Tables

**Table 1:** List of the physicochemical properties calculated and predicted for the designed peptide RiLK1 in comparison with those of the parent AMP 1018-K6.

PARAMETERS	RiLK1 RLKWVRIWRR ^a^	1018-K6V RLIVKVRIWRR ^b^
*Mol weight (Da)*	1468.96	1594.02
*Boman Index (kcal/mol)*	4.70	3.00
*Total net charge*	+5	+5
*Half-life (sec)*	855.71	835.61
*Hydrophobicity*	−0.56	−0.35
*Hydropathicity*	−1.12	0.22
*Amphipathicity*	1.35	1.12
*Hydrophilicity*	0.31	0.14
*Total Trp ratio (%)*	20	8

The conserved amino acid residues of the parent peptide 1018-K6 are highlighted in red. ^a^ This work; ^b^ Ref [14].

**Table 2:** Secondary structure contents of RiLK1 in the absence or presence of SDS (3 mM) determined by BeStSel (Beta Structure Selection) and DichroWeb server.

Incubation Time	BeStSel			DichroWeb CONTIN-LL		
	α-Helix	β-Sheet	Random	α-Helix	β-Sheet	Random
**Without SDS**	1.2%	47.3%	51.5%	4%	35%	61%
**t = 0 with SDS**	7.4%	51.4%	41.3%	22%	38%	40%
**t = 5 min with SDS**	4.4%	53.4%	42.2%	22%	42%	36%
**t = 30 min with SDS**	11%	48.7%	40.3%	25%	38%	37%
**t = 1 h with SDS**	9.8%	51.1%	39.1%	23%	38%	39%
**t = 5 h with SDS**	6.7%	54%	39.3%	19%	43%	38%
**t = 6 h with SDS**	8%	52%	40.1%	19%	44%	37%
**t = 24 h with SDS**	8.4%	53.6%	38%	29%	47%	24%

**Table 3:** Secondary structure contents of RiLK1 at different temperatures in the absence or presence of SDS (3 mM) determined by BeStSel and DichroWeb server.

Temperature (°C)	BeStSel			DichroWeb CONTIN-LL		
	α-Helix	β-Sheet	Random	α-Helix	β-Sheet	Random
**t = 0**	17.9%	37.1%	44.9%	21%	43%	36%
**T = 4 °C**	6.5%	46%	47.5%	25%	37%	38%
**T = 25 °C**	6.8%	49.7%	43.6%	24%	39%	37%
**T = 90 °C**	3%	50.4%	46.7%	6%	52%	42%

**Table 4:** Secondary structure contents of RiLK1 at different pH values determined by BeStSel and DichroWeb server.

pH	BeStSel			DichroWeb CONTIN-LL		
	α-Helix	β-Sheet	Random	α-Helix	β-Sheet	Random
**pH 2 t = 0**	18.5%	37%	44.4%	22%	39%	39%
**pH 2 t = 24 h**	13.2%	42.5%	44.3%	17%	44%	39%
**pH 4 t = 0**	7.7%	39.8%	52.4%	23%	41%	36%
**pH 4 t = 24 h**	13.3%	45.5%	41.2%	24%	41%	35%
**pH 7 t = 0**	15%	39.5%	45.4%	25%	39%	36%
**pH 7 t = 24 h**	15.5%	43.8%	40.8%	19%	44%	37%
**pH 9 t = 0**	13.7%	42%	44.4%	19%	42%	39%
**pH 9 t = 24 h**	13.4%	43.8%	42.9%	29%	47%	24%
**pH 11 t = 0**	14.5%	35.3%	50.2%	16%	31%	53%
**pH 11 t = 24 h**	5.2%	26%	68.8%	14%	33%	53%

**Table 5:** IC_50_ and MBC values of the tested peptides against the bacterial strains.

Strain	RiLK1		1018-K6	
	IC_50_ [µM]	MBC [µM]	IC_50_ [µM]	MBC [µM]
*E. coli*	1.20 ± 0.10	2.0	1.50 ± 0.17	2.0
*L. monocytogenes* (LM3)	0.46 ± 0.01	2.0	0.30 ± 0.02	8.0
*S.* Typhimurium	1.30 ± 0.14	2.5	2.30 ± 0.27	25.0
*S. aureus*	1.98 ± 0.25	16.0	0.92 ± 0.07	16.0

## Supplementary Materials

Supplementary Figure S1 can be found at https://www.mdpi.com/1422-0067/21/18/6952/s1.
