# Supplementary material for: A Safe and Multitasking Antimicrobial Decapeptide: The Road from De Novo Design to Structural and Functional Characterization
Source: Int J Mol Sci. 2020 Sep 22;21(18):6952. doi: 10.3390/ijms21186952 (PMC7555028; doi:10.3390/ijms21186952)
Supplement: Supplementary file 1 [file ijms-21-06952-s001.pdf]

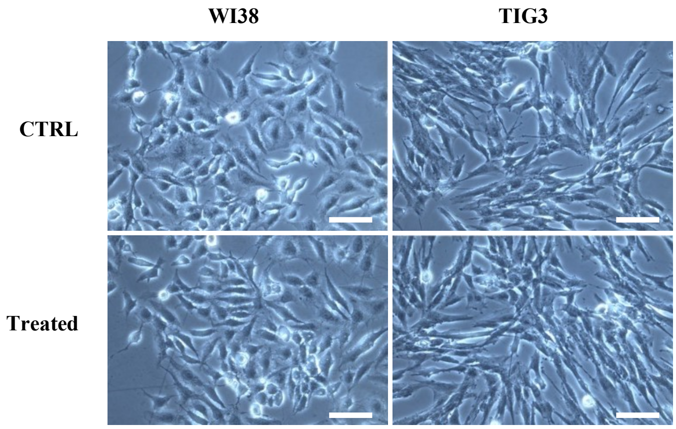

Supplementary Figure 1. Morphological observation of different human cell lines treated with 1018-K6 under phase-contrast microscope. Embryonic (WI38) and fetal (TIG3) lung fibroblastic cell lines were incubated at 37 °C for 24 h in absence (CTRL) or in presence (Treated) of 1018-K6 (10 µM). The microscope images are representative of three independent experiments performed in triplicate. Bar is equal 100 µm.
